# Case Report: Erythema gyratum repens associated with adenomyosis and endometriosis: an immunological twist?

**DOI:** 10.3389/fimmu.2026.1835278

**Published:** 2026-07-10

**Authors:** Emanuele Scala, Donatella Sordi, Arlind Kalaja, Francesca Passarelli, Lidia Francesconi, Filomena Russo

**Affiliations:** 1 Laboratory of Experimental Immunology, Istituto Dermopatico dell’Immacolata, IDI-IRCCS, Rome, Italy; 2 Department of Dermatology, Istituto Dermopatico dell’Immacolata, IDI-IRCCS, Rome, Italy; 3 Dermatological Department, Ospedale Guglielmo da Saliceto, Piacenza, Italy; 4 Pathology Unit, Istituto Dermopatico dell’Immacolata, IDI-IRCCS, Rome, Italy

**Keywords:** adenomyosis, case report, endometriosis, erythema gyratum repens, hysterectomy

## Abstract

A 37-year-old non-Caucasian woman presented with a 2-year history of pruritic, concentric erythematous plaques refractory to multiple topical and systemic therapies. The clinical findings were highly suggestive of erythema gyratum repens (EGR), a rare figurate dermatosis classically associated with paraneoplastic syndromes. Routine laboratory investigations and an autoantibody panel were unremarkable, except for a significantly elevated CA-125 level. A targeted gynecological assessment, prompted by a chronic history of pelvic pain and menorrhagia, identified severe adenomyosis with concomitant endometriosis. Given the severity of her symptoms, the patient underwent a total hysterectomy. Following surgery, the cutaneous eruption resolved completely within 2 months without additional dermatologic intervention. This case broadens the spectrum of non-paraneoplastic conditions associated with EGR and suggests that such eruptions may arise within the context of chronic inflammatory gynecological disease. Clinicians should remain aware of both paraneoplastic and non-paraneoplastic etiologies in patients presenting with EGR to ensure accurate diagnosis, appropriate malignancy screening, and tailored management.

## 1 Introduction

Erythema gyratum repens (EGR) is a rare figurate dermatosis characterized by pruritic, concentric plaques with centrifugal spread. It typically involves the trunk and proximal extremities while sparing the hands, feet, and face (1).

Although most cases are paraneoplastic, EGR has also been reported in non-paraneoplastic settings, including autoimmune disorders, infections, drug reactions, and other benign dermatoses (1, 2).

Here, we report a case of non-paraneoplastic EGR in a patient with severe adenomyosis and endometriosis that resolved following hysterectomy. We also review the spectrum of paraneoplastic and non-paraneoplastic triggers associated with EGR and summarize proposed pathogenetic mechanisms underlying the condition.

## 2 Case presentation

**Figure 1:**
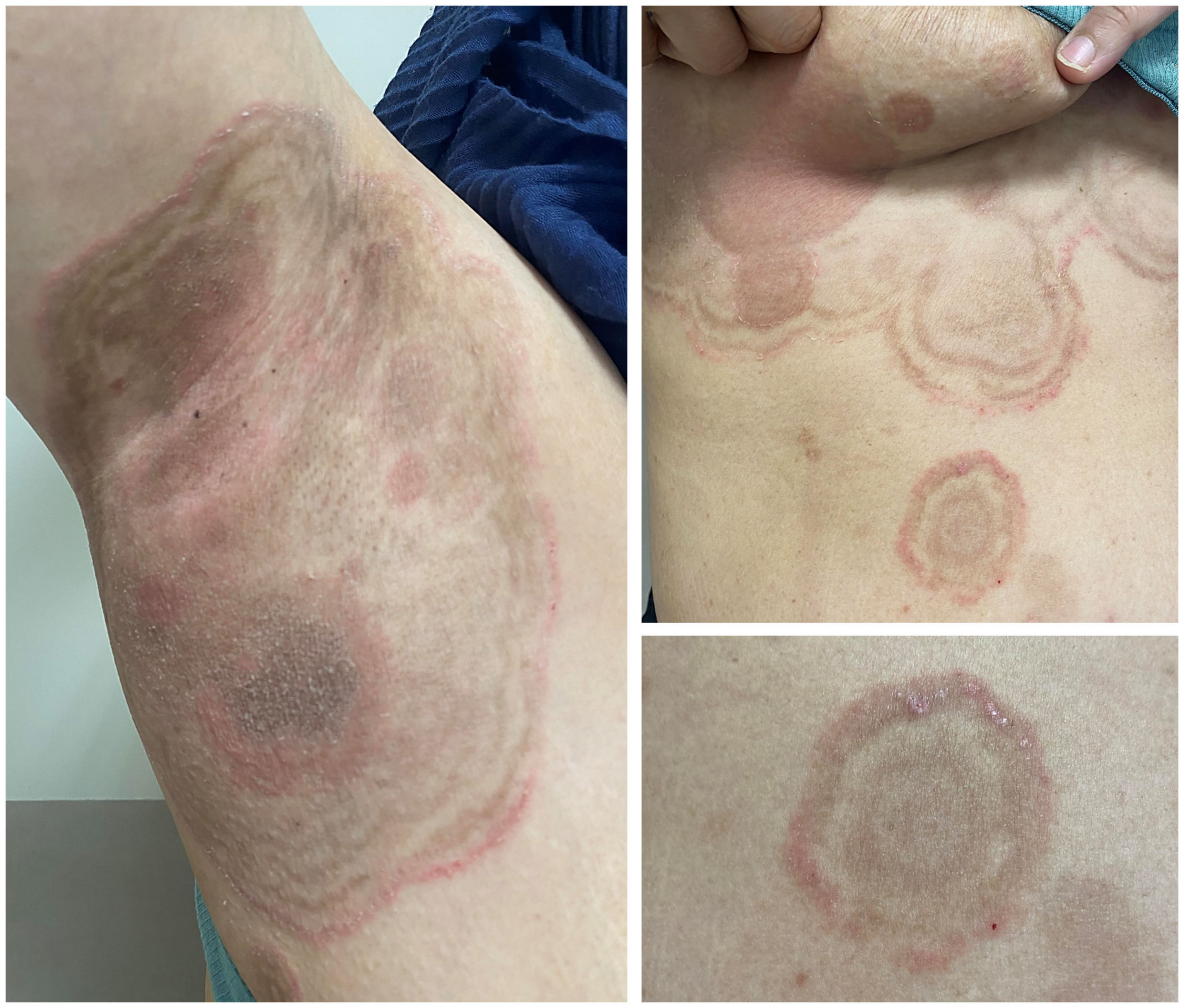
Clinical presentation of erythema gyratum repens on the axillae and trunk, with concentric, erythematous plaques and trailing scale.

A 37-year-old non-Caucasian woman presented to our dermatology clinic with a 2-year history of pruritic, concentric erythematous plaques with trailing scales, involving approximately 20% of her body surface area. The lesions progressively extended from the chest to the inframammary regions, flanks, and axillae (**Figure 1**). The patient reported that the lesions initially appeared as small erythematous patches (1–2 cm in diameter) that gradually coalesced into larger plaques measuring 8–22 cm, with apparent fluctuation in size in relation to her menstrual cycle. She also reported chronic pelvic pain and menorrhagia.

Her medical history included hypertension, type 2 diabetes mellitus, two full-term pregnancies, and one miscarriage. She was a non-smoker, with no relevant occupational exposures or family history of malignancy. Prior empiric treatments included topical clobetasol propionate 0.05% ointment twice daily for 6 weeks, oral prednisone (0.5 mg/kg/day) for 2 weeks, topical ketoconazole 2% cream for 4 weeks, and oral fluconazole 150 mg weekly for 4 weeks, all without clinical improvement.

Although the clinical presentation was highly suggestive of EGR, the differential diagnosis included erythema annulare centrifugum, erythema migrans, necrolytic migratory erythema, psoriasis, pityriasis rubra pilaris, tinea corporis, cutaneous T-cell lymphoma, lupus erythematosus, and drug-induced eruptions. Given that EGR is predominantly a paraneoplastic phenomenon, a definitive differentiation between a paraneoplastic and a non-paraneoplastic etiology could not be made on clinical or histopathological grounds alone. Therefore, an exhaustive oncologic search was required. A stepwise, multidisciplinary diagnostic work-up was initiated to exclude an underlying neoplasm. Laboratory testing, histopathology, and imaging were performed.

Routine blood tests, inflammatory markers, and an autoantibody panel (ANA titer <1:40) were within normal limits. Tumor markers (CA 19-9, CEA, AFP) were within reference ranges, except for an elevated CA-125 level at 181.8 IU/mL (reference <35 IU/mL).

**Figure 2:**
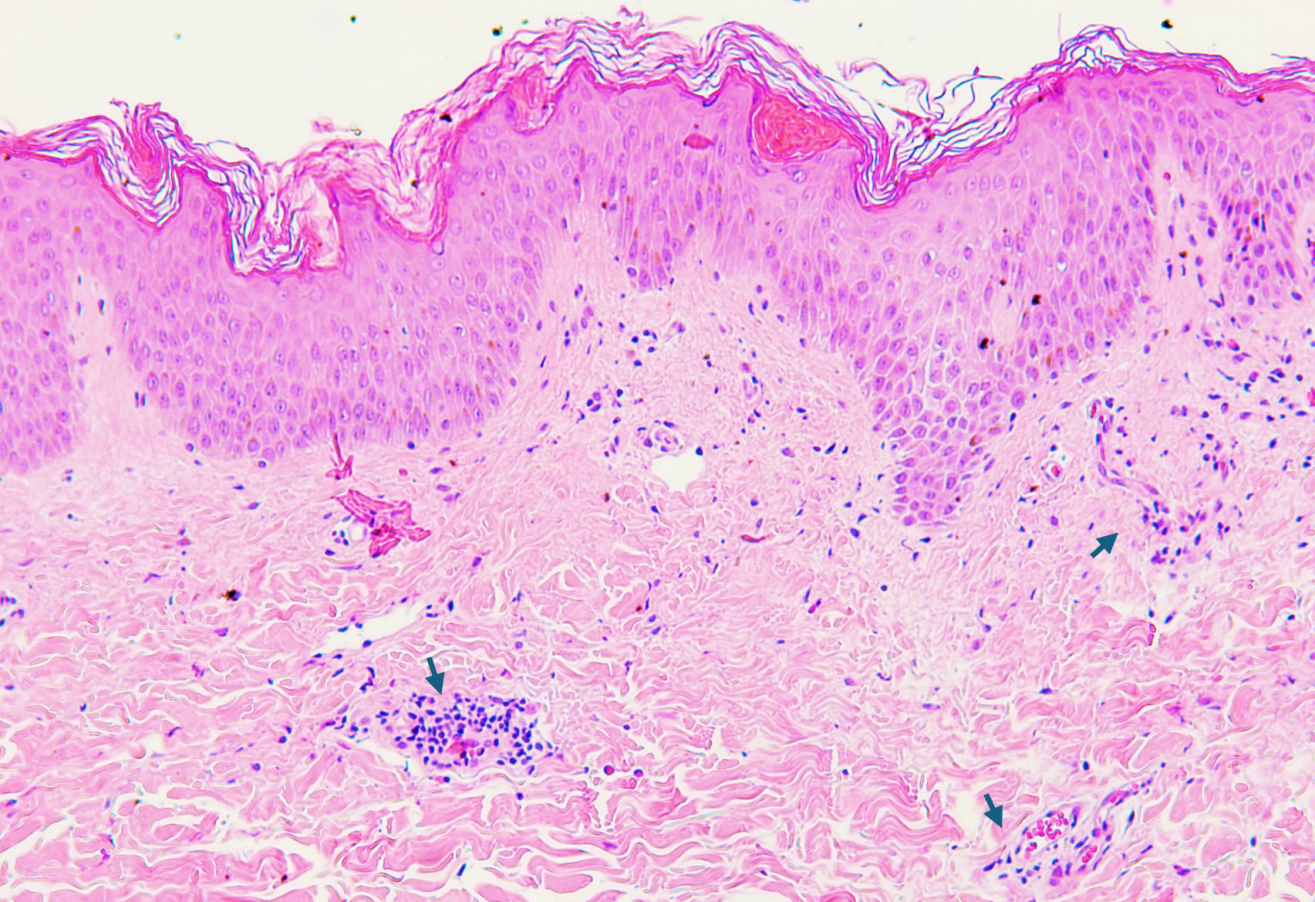
Histopathology showing mild acanthosis, spongiosis, and a sparse lymphocytic infiltrate with scattered eosinophils in the upper dermis. The arrows indicate the perivascular inflammatory infiltrate (hematoxylin and eosin staining, original magnification ×10).

Skin biopsy revealed mild acanthosis and spongiosis with a sparse superficial perivascular lymphocytic infiltrate and scattered eosinophils (**Figure 2**). Direct immunofluorescence performed on three separate sections was negative for IgG, IgA, IgM, and C3. Overall, histopathological findings were nonspecific but supportive of EGR by exclusion. Mycological examination, including direct microscopy and culture, was negative.

Cross-sectional screening imaging, including mammography and contrast-enhanced computed tomography of the chest, abdomen, and pelvis, showed no evidence of malignancy.

In view of persistent pelvic symptoms, a targeted gynecological ultrasound demonstrated an enlarged, globular uterus with heterogeneous myometrial texture, suggestive of adenomyosis. These findings were confirmed by a specialist gynecological assessment, which established a diagnosis of severe adenomyosis with concomitant endometriosis.

**Figure 3:**
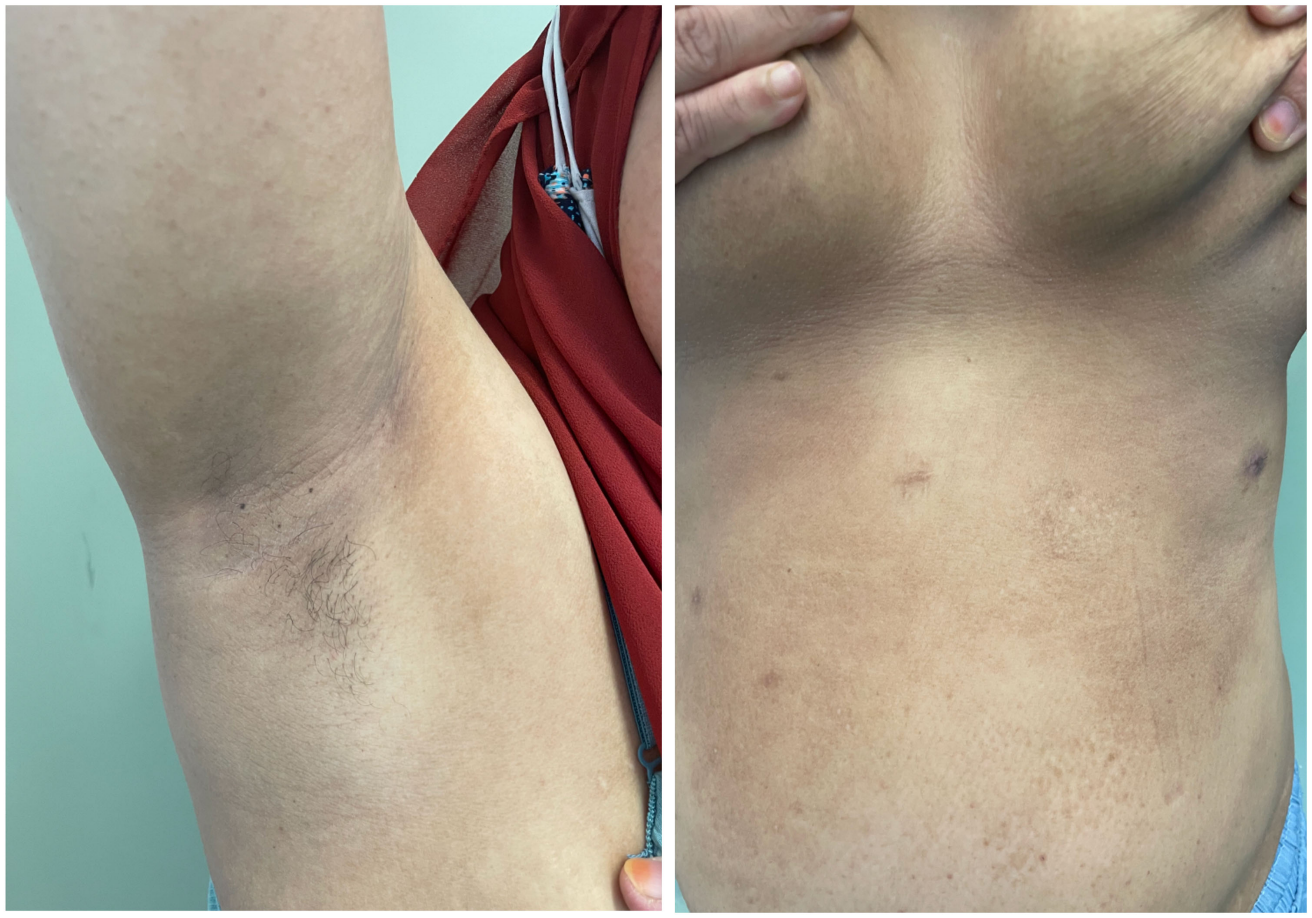
Dermatologic follow-up two months after hysterectomy, showing complete resolution of erythema gyratum repens without further therapy required.

A comprehensive multidisciplinary evaluation involving dermatology, gynecology, radiology, and internal medicine was instrumental in guiding the management strategy. The patient’s menorrhagia and chronic pelvic pain had been severely debilitating and refractory to multiple prior lines of conservative medical management, including oral contraceptives and non-steroidal anti-inflammatory drugs (NSAIDs). Crucially, the decision to proceed with a total abdominal hysterectomy with bilateral salpingectomy was purely therapeutic—aimed at treating her severe, symptomatic adenomyosis and endometriosis—rather than diagnostic, as comprehensive cross-sectional imaging had already ruled out pelvic malignancy. The patient strongly preferred definitive surgical intervention over further conservative or uterine-sparing strategies due to the profound impact of the symptoms on her quality of life. Histopathological examination of the surgical specimen confirmed severe adenomyosis and extensive endometriosis, with no evidence of atypia or malignancy. Strikingly, at the 2-month follow-up after surgery, the cutaneous lesions had completely resolved without additional dermatologic treatment (**Figure 3**). The patient was subsequently lost to follow-up, limiting long-term assessment.

## 3 Discussion

EGR is a rare figurate dermatosis most commonly reported in Caucasian populations (1, 3). It shows a male predominance (male-to-female ratio ≈ 2:1) and typically affects older adults (mean age around 63 years) (1–3). First described by Gammel in 1952, EGR was initially reported in a patient who developed the condition 9 months prior to a diagnosis of breast adenocarcinoma (4). Since then, it has been classically considered a paraneoplastic dermatosis, with associated malignancies reported in approximately 70–82% of cases (1, 3). Nevertheless, an increasing number of non-paraneoplastic cases have been described (1, 3, 5–13), expanding the clinical spectrum (**Table 1**). Reported non-paraneoplastic associations include chronic inflammatory skin diseases (~52%), idiopathic cases (~32%), autoimmune disorders (~8%), systemic infections (~4%), and drug exposures (~4%) (1–3).

**Table 1:** Clinical entities reported in association with EGR.

Category	Entities	References
Malignancies	Lung cancer, esophageal cancer, breast cancer, stomach cancer, genitourinary cancer, lymphoma, multiple myeloma	Gammel JA (4) Silva et al. (2) Boehner et al. (1)
Chronic inflammatory skin diseases	Psoriasis, pityriasis rubra pilaris, ichthyosis	Verma et al. (5) Demonchy (6), Almaani et al. (7) Ridge et al. (8)
Autoimmune disorders	Rheumatoid arthritis, CREST syndrome	Lo Schiavo et al. (9) Rongioletti et al. (3)
Systemic infections	Tuberculosis, *Helicobacter pylori*	Barber et al. (10) Boehner et al. (1)
Drug exposure	Azathioprine, interferon	von Rainer Günther et al. (11) Rongioletti et al. (12)
Immunizations	COVID-19 vaccination	Chiquito et al. (13)

Although these associations are well recognized, the pathogenesis of EGR remains incompletely understood. Proposed mechanisms, mainly derived from paraneoplastic cases, include tumor-driven immune responses with cross-reactivity to epidermal antigens, circulating immune complexes, and antigenic stimulation at the dermoepidermal junction (1, 14). However, these hypotheses do not fully explain the characteristic clinical morphology—rapidly migrating concentric erythematous bands with a “wood-grain” pattern (~1 cm/day)—highlighting the still elusive pathophysiology of the disease. Histopathology remains nonspecific, serving primarily to rule out alternative diagnoses rather than to confirm EGR, thereby reinforcing that the condition remains a clinical diagnosis of exclusion (1).

Our case suggests that EGR may not be confined to older or Caucasian populations with underlying malignancy. In this non-Caucasian woman in her late thirties, no evidence of cancer was found, and the cutaneous lesions resolved within 2 months following a therapeutic total hysterectomy performed for benign, refractory gynecological disease. Although this temporal relationship is notable, causality cannot be definitively established, and the medium-term recurrence risk remains unknown. Limitations of this report include the short follow-up period and the absence of specific immunologic markers directly linking benign gynecological conditions to EGR, making mechanistic interpretations purely hypothesis-generating.

From a pathogenetic perspective, adenomyosis and endometriosis are characterized by immune dysregulation, hormonal influences, and increased pro-inflammatory mediators (15). Adenomyosis has been associated with a relative reduction in regulatory T cells (Tregs), an increased Th17/Treg ratio, and activation of the HMGB1/TLR4 pathway, contributing to a chronic pro-inflammatory environment (15). Endometriosis is characterized by macrophage infiltration, reduced natural killer (NK) cell cytotoxicity, and a cytokine milieu that includes IL-1β, IL-6, TNF-α, IL-10, and TGF-β (15). Biomarkers such as VEGF, MCP-1, and CA-125 have shown diagnostic utility for endometriosis, particularly in relation to the menstrual cycle phase (15). In line with these findings, our patient presented with elevated serum CA-125 and a sparse, non-specific lymphocytic infiltrate on skin biopsy.

Previous studies in EGR have reported immune complex deposition (IgG and C3) and systemic immunologic alterations, including decreased T-cell populations and impaired cell-mediated immunity (16). However, available data remain limited and heterogeneous. Future studies are needed to clarify the precise immunopathogenic mechanisms linking EGR with both paraneoplastic and non-paraneoplastic inflammatory conditions, including endometriosis-related disorders.

## 4 Conclusion

This case expands the spectrum of non-paraneoplastic EGR and suggests a possible, though unproven, association with adenomyosis and endometriosis. While causality cannot be inferred due to the limited follow-up, the case supports the notion that EGR may arise within diverse immunologic or inflammatory contexts. Clinicians should consider both paraneoplastic and non-paraneoplastic etiologies in patients presenting with EGR. Comprehensive, age-appropriate malignancy screening remains essential to exclude underlying neoplasia and ensure timely management. Furthermore, a multidisciplinary approach is crucial in guiding the diagnostic evaluation and therapeutic decision-making, thereby reducing the potential morbidity associated with unrecognized triggers of EGR.

## Data Availability

The datasets presented in this article are not readily available because the authors obtained written informed consent from the patient for the publication of the photograph and medical information in print and online, with the understanding that this information may be publicly available. Requests to access the datasets should be directed to f.russo@idi.it.
